# Supplementary material for: An Electrochemical Impedance Spectroscopy-Based Aptasensor for the Determination of SARS-CoV-2-RBD Using a Carbon Nanofiber–Gold Nanocomposite Modified Screen-Printed Electrode
Source: Biosensors (Basel). 2022 Feb 25;12(3):142. doi: 10.3390/bios12030142 (PMC8945915; doi:10.3390/bios12030142)
Supplement: Supplementary file 1 [file biosensors-12-00142-s001.zip › biosensors-1585339-supplementary.pdf]

biosensors

MDPI

Supplementary material

An Electrochemical Impedance Spectroscopy-Based Aptasensor for the Determination of SARS-CoV-2-RBD Using a Carbon Nanofiber–Gold Nanocomposite Modified Screen-Printed Electrode

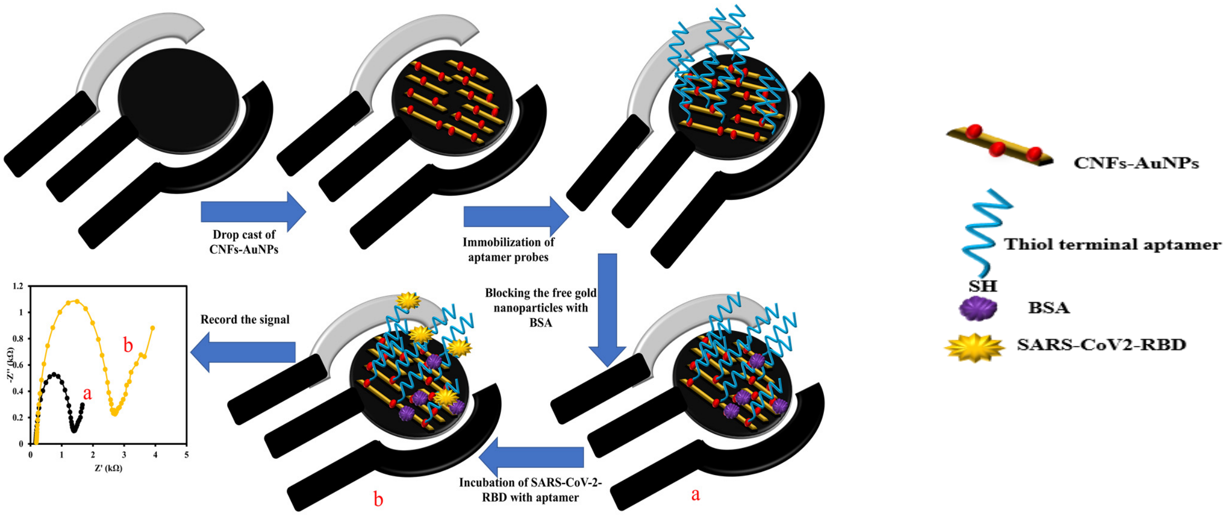

Figure S1. The schematic illustration for the fabrication of the CSPE/CNF–AuNP/Aptamer.

Calculation of the Electroactive Surface Area (A_{eas}), Roughness Factor (RF), and Heterogeneous Electron-Transfer Constant (k_0)

The A_{eas} value for a CSPE and CSPE/CNFs–AuNPs was calculated by using the Randles–Sevčik equation for a quasi-reversible electrochemical process (Equation 1) via drawing the I_{pa} versus the square root of scan rate ($\upsilon^{1/2}$) (Equation 1):

$$I_p^{quasi} = \pm 0.436 \times F \times A_{eas} \times C \times \sqrt{\frac{n \times F \times D \times \upsilon}{R \times T}} \quad (1)$$

where I_p^{quasi} is the peak current; n is the number of electrons (n = 1); F is the Faraday constant (96485 C.mol^{-1}); A_{eas} is the electroactive surface area of the electrode; C is the concentration of $Fe(CN)_6^{3-/4-}$ (5.0×10^{-6} mol.cm^{-3}); D is the diffusion coefficient (7.6×10^{-6} cm^2.s^{-1}); υ is the scan rate (V.s^{-1}); R is the gas constant (8.314 J K^{-1} mol^{-1}); and T is temperature (298 Kelvin). The A_{eas} value for a CSPE and CSPE/CNFs–AuNPs were found to be 0.046 cm^{-2} and 0.251 cm^{-2}, respectively. Additionally, the roughness factor (RF) of the CSPE and the CSPE/CNFs–AuNPs was found to be 0.77 and 4.18, respectively, by using the following equation:

$$RF = \frac{A\ eas}{A\ gsa} \quad (2)$$

where the geometric surface area (A_{gsa}) of the CSPE is 0.06 cm^{-2}. It indicates that CNFs–AuNPs improved the electroactive surface area of the screen-printed electrode dramatically.

The heterogeneous electron-transfer constant (k_0) was also obtained by using the Nicholson equation (Equation 3) for a quasi-reversible system:

$$\psi = k_0 \left[\frac{\pi \times D \times n \times F \times \upsilon}{R \times T}\right]^{-0.5} \tag{3}$$

where ψ is a kinetic parameter; ν the scan rate in $mV.s^{-1}$; F the Faraday constant of 96,485 $C.mol^{-1}$; R the universal gas constant of 8.31 $J.K^{-1}.mol^{-1}$; and T the temperature in Kelvin. To find the k_0, the Ψ value must be first to be found. The Ψ value can be obtained by using the equation below (Equation 4):

$$\Psi = \frac{(-0.6288 + 0.0021n \times \Delta Ep)}{(1 - 0.017n \times \Delta Ep)} \tag{4}$$

where ΔE_p is the difference between the potential of the anodic peak and the potential of the cathodic peak. At a scan rate of 10 $mV.s^{-1}$, the ΔE_p of $Fe(CN)_6^{3-/4-}$ at a CSPE and CSPE/CNFs–AuNPs were 234 $mV.s^{-1}$ and 84 $mV.s^{-1}$, respectively. Ψ values for CSPE and CSPE/CNFs–AuNPs were then found to be 0.046 and 1.05, respectively, using the Lavagnini equation. Additionally, the K_0 values for a CSPE and CSPE/CNFs–AuNPs were found to be 0.004 $cm.s^{-1}$ and 0.11 $cm.s^{-1}$, respectively, by using Nicholson equation (Equation 3). It indicates that the CNF–AuNP nanocomposite increased the electron transfer rate of $Fe(CN)_6^{3-/4-}$ to the surface of the electrode.

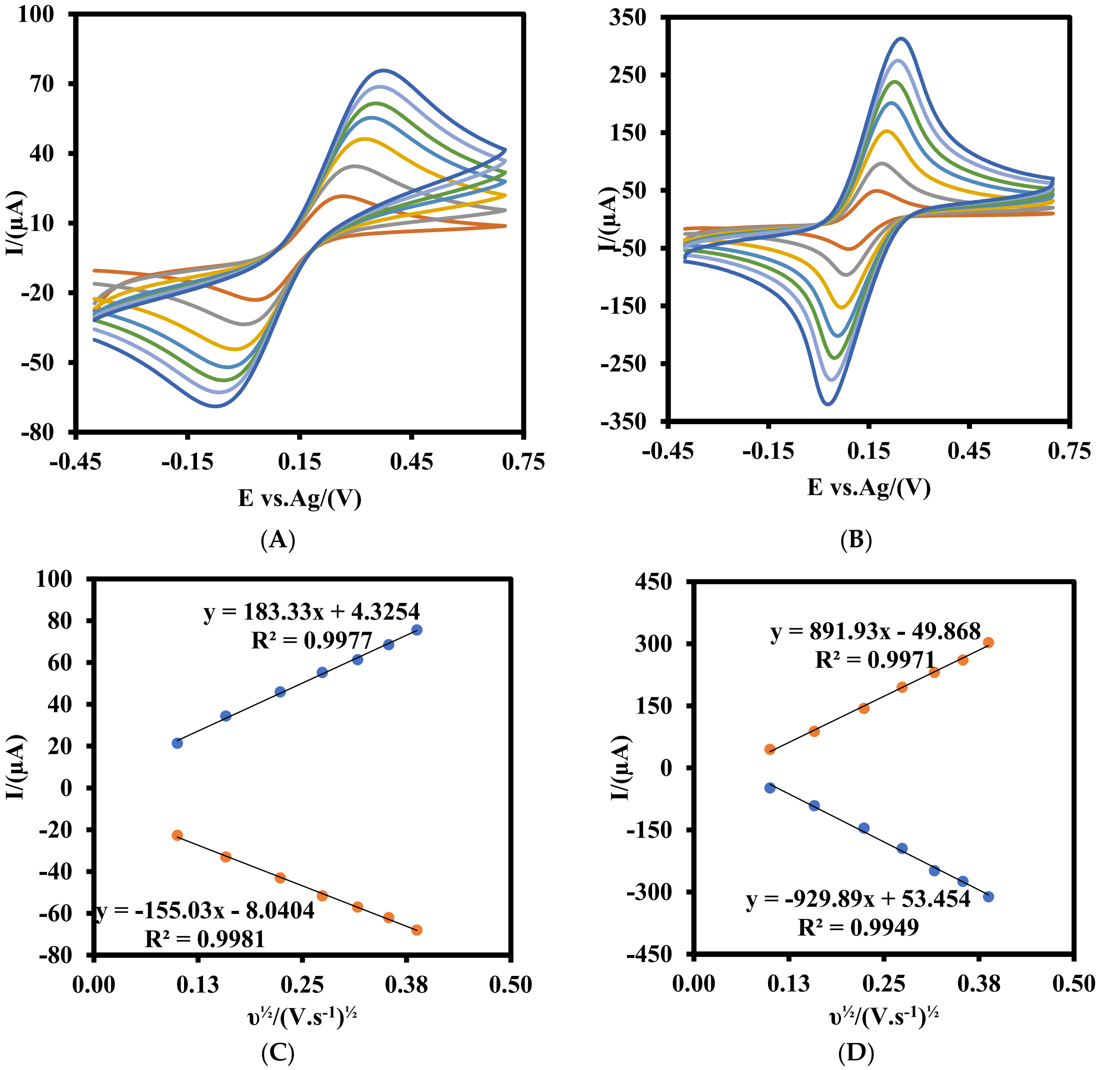

Figure S2. (**A**) CVs of the CSPE and (**B**) CSPE/CNFs–AuNPs in 5.0 mM $Fe(CN)_6^{3-/4-}$ solution (0.1M PBS, pH 7.4) at various scan rates (0.01. 0.025, 0.05, 0.075, 0.1, 0.125, and 0.15 $V.s^{-1}$ from inner to outer). The plot of the anodic peak current (I_{pa}) and cathodic peak current (I_{pc}) versus the square root of scan rate ($\nu^{½}$) for (**C**) CSPE and (**D**) CSPE/CNFs–AuNPs.

Study the Surface Coverage of the Immobilized Aptamer

The surface coverage of the aptamer ($\Gamma_{aptamer}$) on the surface of the electrode was obtained according to the electrostatic interaction between $Ru(NH_3)_6^{3+}$ as a positively charged redox probe and the negatively charged phosphate backbone of aptamer [5]. To this end, the CSPE/CNF–AuNP/Aptamer was first immersed in a $Ru(NH_3)_6^{3+}$ solution (0.1 M PBS, pH 7.4) for 30 min. Then, the aptasensor was washed several times and immersed in 0.1 M PBS (pH 7.4) to record the CVs. As shown in Figure S3A, before the immobilization in the $Ru(NH_3)_6^{3+}$ solution (a), there were not any redox peaks in the CV of the CSPE/CNF–AuNP/Aptamer. However, after the immobilization of the aptasensor in the $Ru(NH_3)_6^{3+}$ solution (b), a couple of well-defined redox peaks of $Ru(NH_3)_6^{3+}$ were observed.

To know the surface coverage of the aptamer probe on the aptasensor, the CVs of the immobilized $Ru(NH_3)_6^{3+}$ on the CSPE/CNF–AuNP/Aptamer were recorded at different scan rates (ν) from 0.01 to 0.2 $V.s^{-1}$ (Figure S3B). The linear dependence of I_{pa} and I_{pc} on ν displays a surface-confined electron transfer process (Figure S3C).

The surface coverage of $Ru(NH_3)_6^{3+}$ ($\Gamma_{Ru(NH3)6]3+}$) can be obtained by the following Equation 5:

$$I=9.35\times10^5\times n\times A_{eas}\times\Gamma_{Ru(NH3)6^{3+}}\times\upsilon \quad (5)$$

where n is the number of released electrons by $Ru(NH_3)_6^{3+}$ during the electrochemical process (n = 1 for $Ru(NH_3)_6^{3+}$). The average $\Gamma_{Ru(NH3)6^{3+}}$ value was calculated to be 898 $pmol.cm^{-2}$.

There are 51 nucleotide bases in the structure of the aptamer probe. As a nucleotide base can interact with $Ru(NH_3)_6^{3+}$, therefore, the $\Gamma_{aptamer}$ value can be obtained by using following equations:

$$\Gamma_{Aptamer}=\Gamma_{Ru(NH3)6^{3+}}\times\frac{z}{m} \text{ (eq 6) and } \Gamma_{Aptamer}=\Gamma_{MB}\times\frac{z}{m}\times N_A \quad (6)$$

where z is the charge of the adsorbed $Ru(NH_3)_6^{3+}$ (z = 3); m is the number of nucleotides that can interact with the adsorbed probe (m = 51); and N_A is Avogadro's number (6.022×10^{23} $molecules.mol^{-1}$). The $\Gamma_{aptamer}$ value was calculated to be 52.8 $pmol.cm^{-2}$ (eq 6) or 3.18×10^{13} $molecules.cm^{-2}$ (Equation 7). The $\Gamma_{Aptamer}$ value was higher than the previously reported aptasensor for the SARS-CoV-2-RBD [7], indicating that the CNF–AuNP nanocomposite provided a high-surface area for the immobilization of the aptamer probe.

The K_s value of $Ru(NH_3)_6^{3+}$ on the CSPE/CNF–AuNP/Aptamer was obtained by using Laviron's formula (Equation 7) for the surface-controlled electrochemical system ($\Delta Ep <$ 200 mV, α = 0.5) [8]:

$$K_s=\frac{m\times n\times F\times\upsilon}{R\times T} \quad (7)$$

where m is the parameter related to the peak potential separation; n the number of electrons involved in the reaction; ν the scan rate; F the Faraday constant of 96,485 $C.mol^{-1}$; R the universal gas constant of 8.31 $J.K^{-1}.mol^{-1}$; and T the temperature in Kelvin. The K_s value was calculated to be 1.5 s^{-1}. The high value of K_s shows that the electron transfer of self-assembled $Ru(NH_3)_6^{3+}$ on the proposed aptasensor has good reversibility.

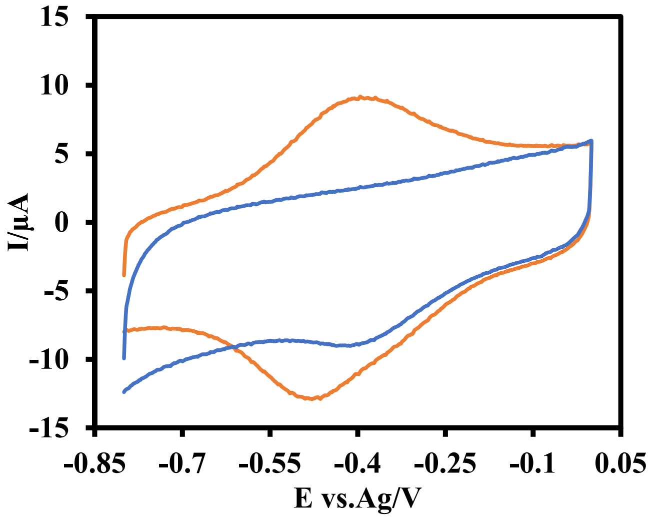

(**A**)

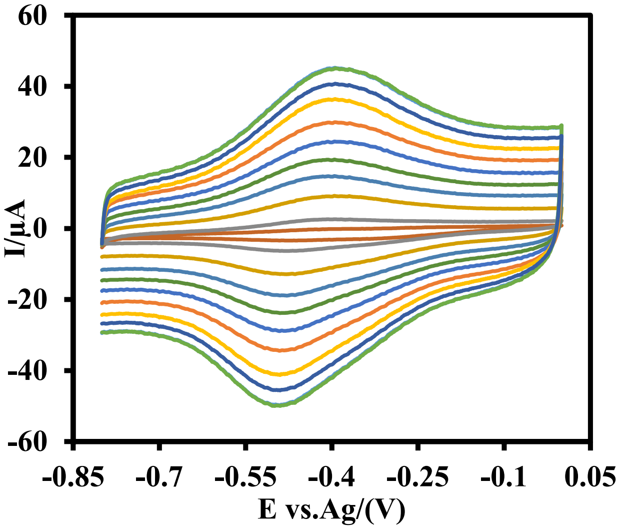

(**B**)

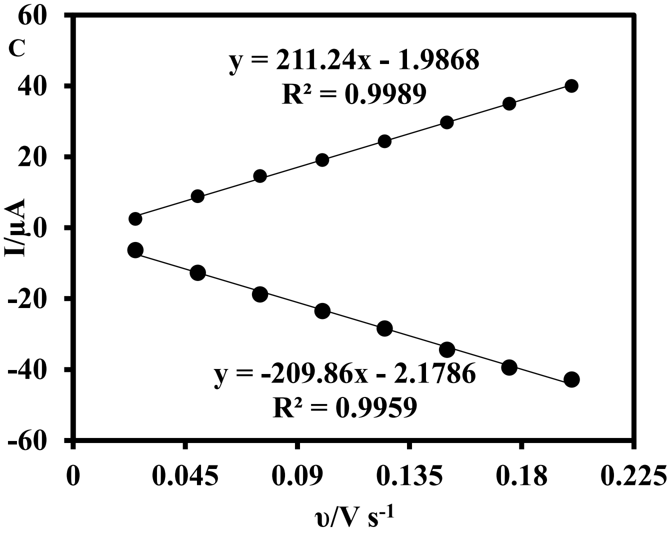

(**C**)

Figure S3. (**A**) CVs of a CSPE/CNF–AuNP/Aptamer before (a) and after (b) the immobilization of $Ru(NH_3)_6^{3+}$. (**B**) CVs of the CSPE/CNF–AuNP/Aptamer–$Ru(NH_3)_6^{3+}$ in a PBS (0.1 M, pH 7.4) at various scan rates (0.01. 0.025, 0.05, 0.075, 0.1, 0.125, 0.15, 0.175, and 0.2 $V.s^{-1}$ from inner to outer). (**C**) The plot of the anodic peak current (I_{pa}) and cathodic peak current versus scan rate (ν).

Table S1. The details of the EIS spectrum related to Figure 3.

Electrode	R1	R2	W1	P1	n
CSPE	192.2	3048.2	976.81	5.28e-7	0.918
CSPE/CNFs–AuNPs	93.2	135.79	248.7	1.6e-4	0.368
CSPE/CNF–AuNP/Aptamer	165.12	1186.6	450.2	6e-7	0.9

CSPE/CNF–AuNP/Aptamer/SARS-CoV-2-RBD	188.8	2432	751.9	5.4e-7	0.919

Optimization of Effective Parameters on the Response of the CSPE/CNF–AuNP/Aptamer

As shown in Figure S4A, the R_{ct} of the CSPE/CNF–AuNP/Aptamer increased as the concentration of the immobilized aptamer probe increased from 30 to 120 nmol and then decrease as it increased to 150 nmol. This can be explained due to the increase in the repulsion force between the negatively charged nucleotide acids of an aptamer probe and other aptamers in a high concentration. This repulsion interaction inhibited the interaction of the thiol-terminal aptamer probes with AuNPs. Therefore, 120 nmol of thiol-terminal aptamer probes was chosen as an optimum concentration of the aptamer probe for the fabrication of the aptasensor.

The effect of time and pH on the incubation process of SARS-CoV-2-RBD on aptasensor was also studied (Figure S4B, S4C).

As shown in Figure S4B, the response of the aptasensor decreased by increasing the incubation time from 10 min to 40 min and then remained unchanged at a longer incubation time, indicating that the SARS-CoV-2-RBD/Aptamer complex formation on the electrode surface reached a saturation level. During this process, the CSPE/CNF–AuNP/Aptamer was immersed in 0.1 M PBS (pH 7.4) containing 2.0 nM of SARS-CoV-2-RBD and then the solution was stirred.

The pH effect of the incubation solution on the response of the CSPE/CNF–AuNP/Aptamer was also studied (Figure S4C). As the pH of the incubation solution containing 2.0 nM of SARS-CoV-2-RBD increased from 5.4 to 7.4, the R_{ct} of the CSPE/CNF–AuNP/Aptamer to 2.0 nM of SARS-CoV-2-RBD increased and reached the maximum value at pH 7.4, but decreased as the incubation pH increased to 8.4.

This can be explained by two reasons. In the acidic or basic solution, the aptamer probe would be either denatured from its hairpin-shaped structure to a non-shape structure or its nucleotides would be the protonate/deprotonate. It caused the dysfunction of the aptasensor. Additionally, denaturation could happen for the SARS-CoV-2-RBD molecule in the acidic or basic solution and then changed its structure. Therefore, the denaturation of SARS-CoV-2-RBD could not interact with the aptamer. Therefore, the incubation time of 40 min and incubation pH of 7.4 were chosen as the optimum conditions throughout the rest of the measurements in this work.

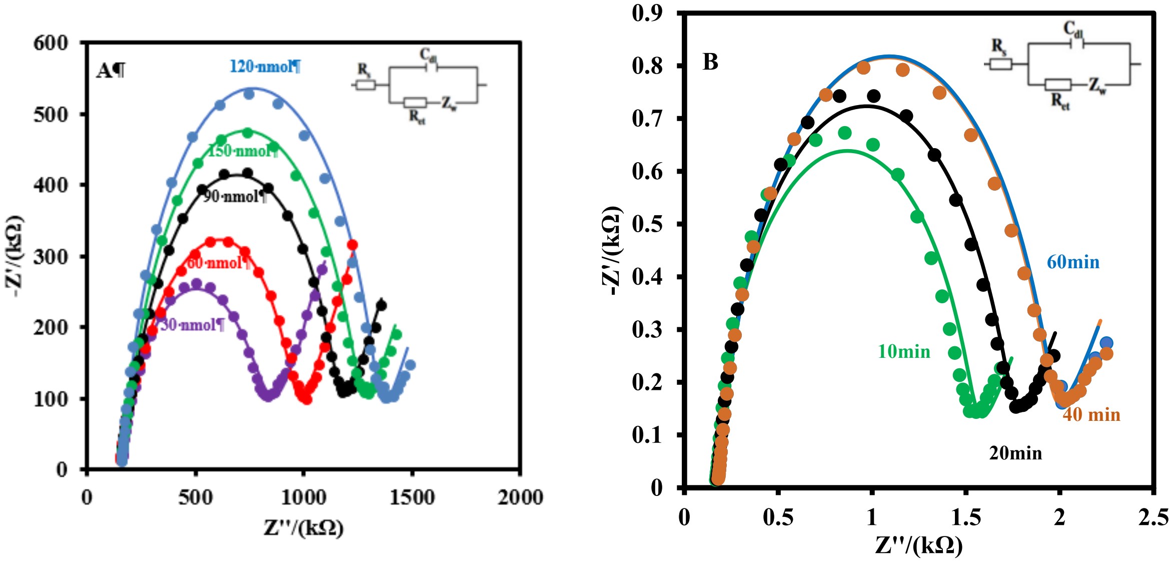

(**A**) (**B**)

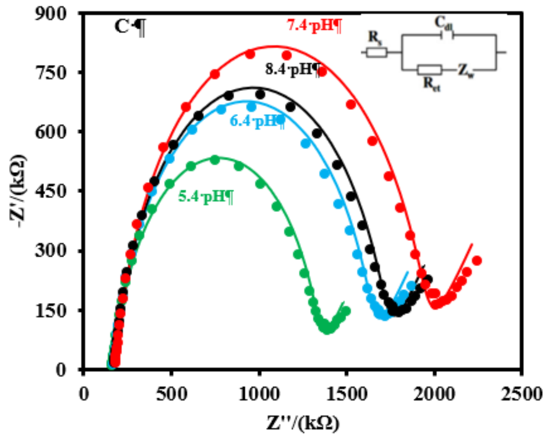

(**C**)

Figure S4. (**A**) Effect of the aptamer probe concentration, (**B**) incubation time, and (**C**) pH on the response of the aptasensor. EIS signals were recorded at 5.0 mM Fe(CN)63−/4− solution (0.1 M PBS, pH 7.4). The equivalent electric circuit is compatible with the Nyquist diagrams. Rs: Solution resistance; Rct: Charge transfer resistance; Cdl: Double layer capacitance; Zw: Warburg impedance. The AC amplitude was 10 mV, DC potential was 0.13 V, and the frequency range was 100 kHz -0.1 Hz.

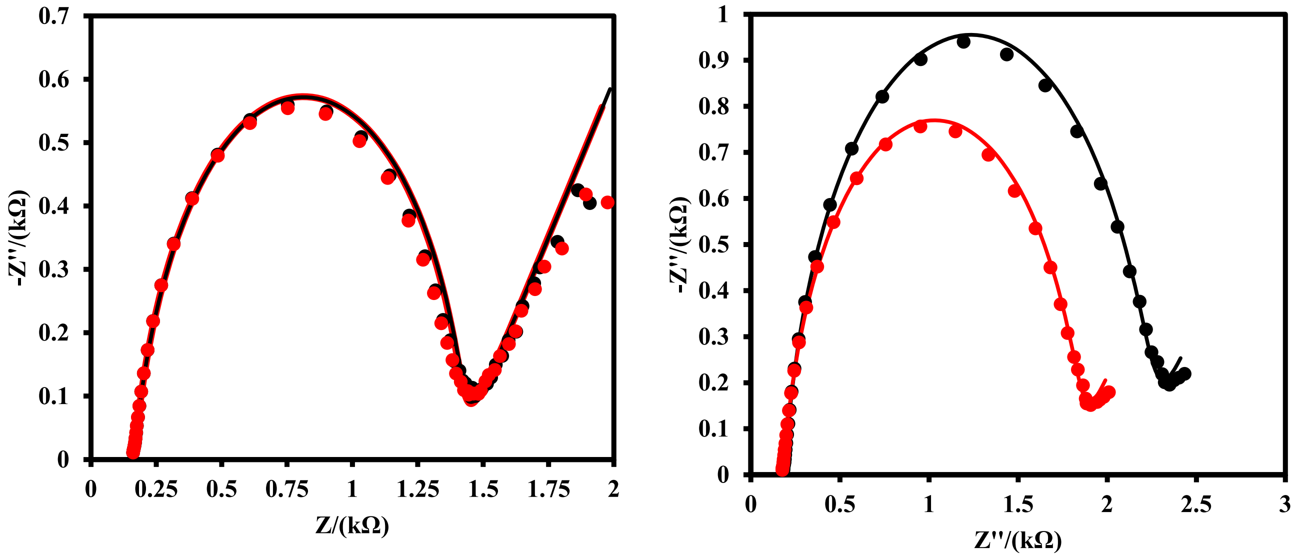

(A) **(B)**

Figure S5. (**A**) EIS of the CSPE/CNF–AuNP/Aptamer in the absence (blue curve) and presence of 50 µL of saliva (red curve). (**B**) EIS of the CSPE/CNF–AuNP/Aptamer to 1 nM SARS-CoV-2 RBD (red curve) and 8 nM SARS-CoV-2 RBD (black curve) that were fabricated in the 2-fold diluted saliva sample. EIS spectrums were recorded in 5.0 mM $Fe(CN)_6^{3-/4-}$ solution (0.1 M PBS, pH 7.4). The equivalent electric circuit is compatible with the Nyquist diagrams. R_s: Solution resistance; R_{ct}: Charge transfer resistance; C_{dl}: Double layer capacitance; Z_w: Warburg impedance. The AC amplitude voltage was 10 mV, DC voltage was 0.13 V, and the frequency range was 100 kHz–0.1 Hz.

References

1. Faulkner, L.R.; Bard, A.J. *Electrochemical method: Fundamentals and applications (second ed.)*. Wiley, New York: 2001.
2. Festinger, N.; Morawska, K.; Ivanovski, V.; Ziąbka, M.; Jedlińska, K.; Ciesielski, W.; Smarzewska, S. Comparative electroanalytical studies of graphite flake and multilayer graphene paste electrodes. *Sensors* **2020**, *20*.

3. Nicholson, R.S. Theory and application of cyclic voltammetry for measurement of electrode reaction kinetics. *Anal. Chem.* **1965**, *37*, 1351-1355.
4. Lavagnini, I.; Antiochia, R.; Magno, F. An extended method for the practical evaluation of the standard rate constant from cyclic voltammetric data. *Electroanalysis* **2004**, *16*, 505-506.
5. Rafiee-Pour, H.-A.; Behpour, M.; Keshavarz, M. A novel label-free electrochemical mirna biosensor using methylene blue as redox indicator: Application to breast cancer biomarker mirna-21. *Biosens. Bioelectron.* **2016**, *77*, 202-207.
6. Steel, A.B.; Herne, T.M.; Tarlov, M.J. Electrochemical quantitation of DNA immobilized on gold. *Anal. Chem.* **1998**, *70*, 4670-4677.
7. Amouzadeh Tabrizi, M.; Nazari, L.; Acedo, P. A photo-electrochemical aptasensor for the determination of severe acute respiratory syndrome coronavirus 2 receptor-binding domain by using graphitic carbon nitride-cadmium sulfide quantum dots nanocomposite. *Sens. Actuators B Chem.* **2021**, *345*, 130377.
8. Laviron, E. General expression of the linear potential sweep voltammogram in the case of diffusionless electrochemical systems. *J. Electroanal. Chem. Interf. Electrochem.* **1979**, *101*, 19-28.
